# Impact of human activities on changes of ichthyofauna in Dongjin River of Korea in the past 30 years

**DOI:** 10.1080/19768354.2017.1330223

**Published:** 2017-05-25

**Authors:** Myeong-Hun Ko, Ye-Seul Kwan, Won-Kyung Lee, Yong-Jin Won

**Affiliations:** a Division of EcoScience, Ewha Womans University, Seoul, South Korea; b Nakdonggang National Institute of Biological Resources, Sangju, South Korea; c Department of Life Science, Ewha Womans University, Seoul, South Korea

**Keywords:** Icthyofauna, fish community, Dongjin River, exotic species, artificial water diversion tunnels

## Abstract

Ichthyofauna and fish community were investigated at 17 representative stations of the Dongjin River drainage system from spring to fall in 2014. The survey resulted in a list of 53 species belonging to 14 families structured into 4 distinctive parts along the river: uppermost-stream, upper-stream, mid-stream, and lower-stream. Comparison of species lists with 30-year interval exhibited significant decreases in peripheral freshwater fishes, Acheilognathinae, endemic, and indigeneity species, but increases in exotic, epipelagic, and lentic species. Moreover, in the estuary of the Dongjin River drainage system, peripheral freshwater fish species were replaced by pure freshwater fish species due to the Saemangeum sea-wall project. In the upper region of the river, introduced eight alien species from Seomjin River via water diversion tunnels. In the mid-lower region, the construction of floodgates and numerous small weirs caused expansion of lentic water areas, facilitating the spread of problematic exotic species such as *Micropterus salmoides*, *Lepomis macrochirus*, and *Carassius cuvieri*. Also, water deterioration in this region resulted in an increase of tolerant species and a decrease of sensitive and endemic species. Our results suggest that a recovery strategy for a healthy ecosystem in the Dongjin River drainage system should reflect this compartmentalized cause and effect on the changes of icthyofauna.

## Introduction

Natural distribution of temperate fishes is affected by multiple factors, including Pleistocene events, zoogeographic barriers, physiological factors, and biological interactions (Moyle & Cech 2000). However, recent human activities have become the main causes of the decrease in biodiversity, changes in community structure, and extinction of many aquatic species (McKinney 2002; Dudgeon et al. 2006; Leprieur et al. 2011; Toussaint et al. 2014). Rapid industrialization and modernization of South Korea since the 1960s have brought in parallel pollution and construction of many large dams, weirs, and sea walls in rivers. These anthropogenic changes have caused severe deterioration of water quality as well as alteration in flows of rivers and streams in many places of Korea (Kwater 2007, Jang et al. 2010). Additionally, introduction of exotic fishes, amphibians, and reptiles has disturbed the aquatic ecosystem of this country (Kim et al. 1996, Bang et al. 2011).

Health assessment of the river has been dependent on chemical factors, such as organic, toxic, and nutrient materials. Recently, however, biological and physical factors are added in assessment of the river health (Barbour et al. 1999). In particular, biological assessment since the development of the index of Biological Integrity (IBI), many countries now assess the river health using fish, benthic invertebrates, attached algae, and waterside vegetation (Oberdorff & Hughes 1992; Lee & An 2014).

**Figure 1.**
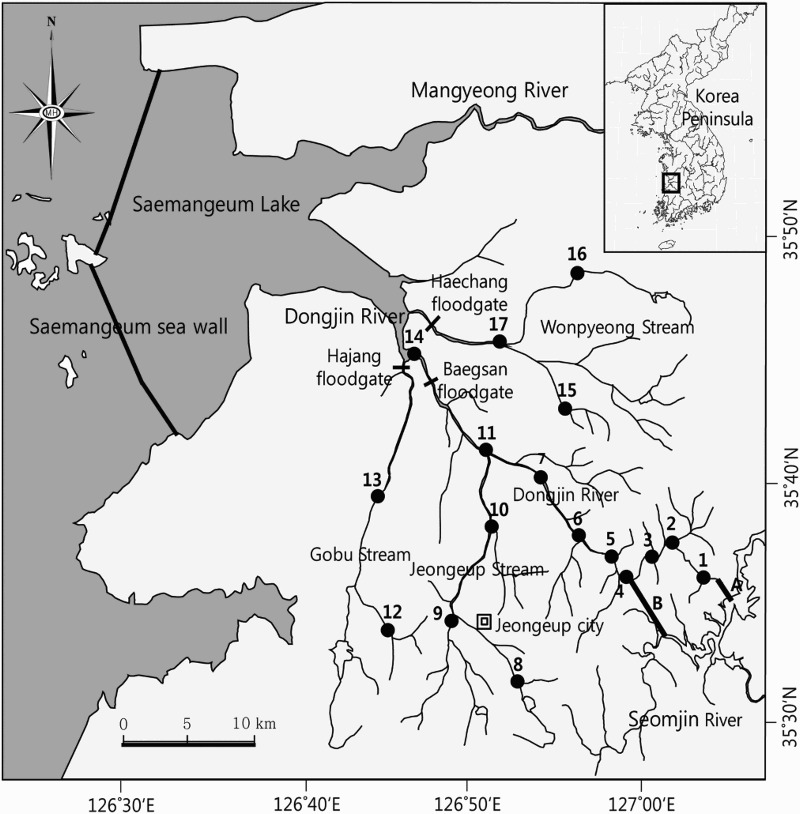
Study stations of the Dongjin River drainage system, Jeollabuk-do, Korea, 2014. The thick black lines (A and B) represent two tunnels connecting the waters of the Dongjin River and Seomjin River (Lake Okjeong).

The Dongjin River drainage system (Figure 1) is located at the southernmost region. Like other rivers in Korea, the Dongjin River has been affected by diverse human activities, some of which are unique to this river, while others are common to all rivers. The source of water begins from Naejang Mountain on Noryeong mountain range. The river length is 51.0 km, and its major tributaries are Jeongeup, Gobu, and Wonpyeong Streams. This river flows on low-altitude plains in its western side, generating highly productive rice paddy fields in Korea (Kwater 2007). In the upper-stream of Dongjin River, river diversion tunnels were built in 1931 and 1965 to utilize the altitude difference of the neighboring Seomjin River and Dongjin River. Since then, these tunnels were used to produce electricity and supply farming water to southwestern plains of Korea (Kwater 2007). As expected, inadvertent inflow of fishes from the Seomjin to Dongjin River has been reported (Kim & Lee 1984). In 2006, the Saemangeum sea wall (33 km) surrounding the estuaries of the Mangyeung and Dongjin Rivers were completed as a part of the Saemangeum Reclamation Project, the largest land reclamation project in the world (Rogers et al. 2006). Completion of the Samangeum dikes at the estuary of Dongjin River has caused rapid habitat alteration (Park et al. 2013). Following this event, introduction and dispersal of exotic fish species such as *Lepomis macrochirus* and *Micropterus salmoides* have been reported (Kim & Lee 1984; Kim 2000).

Given these most notable and unique environmental changes in the Dongjin River, the objectives of this study were: (i) to present recent investigation results of ichthyofauna, the fish community and river health assessment in the entire Dongjin River in 2014, (ii) to compare these results with the results of previous investigations completed by Kim and Lee (1984), Kim (2000) and Kim et al. (2009), and (iii) to discuss the causes and changes in the past 30 years.

## Materials and methods

### Study area and survey period

A total of 17 sites along the entire drainage system of the Dongjin River were selected (Figure 1) according to the areas studied by Kim and Lee (1984) and Kim (2000). In addition, sites were selected at intervals of 3–5 km upstream of the Dongjin River where the fish from the Seomjin River would appear frequently. Ichthyofauna and habitat environments of these sites were investigated in three seasons, spring (7–10 April 2014), summer (6–9 July 2014), and fall (5–8 October 2014). Winter hibernation season was excluded from this study.

### Habitat environment

To obtain hydrological environment data, river width, water width, depth, stream order, altitude, and bottom structures were investigated. River and water widths were measured using a binocular telescope (Yardage pro Tour XL, BUSHNELL, Japan). Water depth was measured using a measuring stick. Physicochemical parameters including water temperature, conductivity, dissolved oxygen (DO) level, salinity, and pH were measured using a digital temperature indicator (T-250A, ASAHI, Japan) and a MultiParameter water quality meter (HI-9828, Romania).

### Taxon sampling and identification

For quantitative investigation, casting net (mesh 6 × 6 mm) was used 10 times, and a skimming net (4 × 4 mm) was used at 200 m section for 40 min at each sampling site. Collected samples were identified and counted. They were released shortly after to conserve the ecosystem. Identification of fish followed that of Kim and Park (2007).

### Similarity analysis and river health assessment

To quantify the compositional similarity between two different sites, the Bray–Curtis similarity was calculated using Primer 5.0 (PRIMER-E Ltd, UK). For this analysis, our field data of the list of species and the abundance of each species at each different site were used. River health assessment was carried out based on the revised model of multivariate matrix IBI developed in the United States (Karr 1981; Barbour et al. 1999), to fit the circumstances of Korea (MEK 2007; NIBR 2015). This model consists of a total of eight-matrix system, and each matrix represents the following: *M*_1_ (number of domestic species), *M*_2_ (number of rapid benthic species), *M*_3_ (number of sensitive species [SS]), *M*_4_ (individual rate of tolerant species), *M*_5_ (omnivores individual ratio), *M*_6_ (insectivores individual ratio of domestic species), *M*_7_ (number of individuals of domestic species), *M*_8_ (abnormal individuals ratio). Matrices *M*_1_, *M*_2_, *M*_3_, and *M*_7_ were assessed differently according to their stream order. Calculated values for each matrix were summed up and assessed into four levels, Excellent (87.5–100), Good (56.2–87.5), Fair (25.0–56.2), or Poor (0–25.0) (NIBR 2015).

### Correlation analysis

Correlation between physicochemical parameters (temperature, DO level, conductivity, salinity, and pH) and habitat characteristics (number of species, number of individuals, SS, tolerant species, and river health assessment) of fishes at each site was analyzed using SPSS 21.0 (IBM, USA).

## Results

### Hydrological and physicochemical environments

From April to September, massive amounts of water influx from the Seomjin River to St. 1 and 4 were detected, with little or none detected from October to March. Lower-stream stations (St. 11, 13, 17) of the Dongjin River showed deep lentic water without riffles due to the construction of floodgate. The water level of the river was high during the farming season (April to September) because the floodgate was closed. However, the water level was rapidly decreased in other seasons when the sluice was open. The water temperature of upper-stream stations (St. 1–4) was lower than that of the middle and lower-stream stations by 3–12°C. Conductivity was low in upper mid-stream stations (St. 1–8) (100–150 µs/cm) but relatively high in mid-lower-stream stations (St. 9–17) (200–600 µs/cm). DO level in upper mid-stream stations (St. 1–8) (9–11 mg/L) was higher than that of mid-lower stations (St. 9–17) (7–10 mg/L). However, the pH value did not differ among stations. In addition, most stations had pure freshwater streams with salinity values lower than 0.3‰ (Table 1).

**Table 1.** Hydrological and physicochemical environments at the study stations in the Dongjin River drainage system, Jeollabuk-do, Korea, from spring to fall, 2014.

St.	River width (m)	Water width (m)	Water depth (m)	Altitude (m)	Stream order	Bottom structure (%)						Water temperature (°C)	Conductivity (μs/cm)	DO (mg/ℓ)	pH
						M	S	G	P	C	B				
1	30–40	10–20	0.5–1.0	95	2	5		15	20	50	10	14.8 ± 2.73	131 ± 20	9.8 ± 0.38	6.4–7.1
2	60–80	10–30	0.3–1.0	56	3		30	40	20	10		17.0 ± 3.08	140 ± 18	10.6 ± 0.15	6.4–7.1
3	50–70	30–40	0.3–1.0	42	3		10	20	50	20		17.6 ± 2.50	133 ± 14	10.6 ± 1.03	6.4–7.1
4	100–120	20–50	0.5–1.5	36	3		20	10	30	30	10	18.4 ± 2.65	133 ± 12	9.5 ± 1.01	6.5–7.1
5	110–120	40–70	0.3–1.2	23	3		20	40	20	10	10	18.4 ± 3.09	140 ± 15	10.4 ± 2.27	6.5–7.1
6	130–150	10–30	0.3–1.2	18	4		20	70	10			18.4 ± 2.73	171 ± 54	9.4 ± 1.45	6.4–7.1
7	150–200	50–100	0.3–1.5	10	4	40	30			10	20	18.1 ± 2.79	125 ± 22	8.9 ± 0.98	7.0–7.1
8	80–100	5–10	0.3–1.0	77	3			10	10	60	20	18.3 ± 6.67	115 ± 22	10.7 ± 0.68	6.8–7.1
9	120–150	30–70	0.3–1.2	27	4	10	20	30	10	20	10	20.0 ± 6.33	281 ± 43	9.1 ± 1.69	6.4–7.0
10	90–100	20–30	0.5–1.0	10	4	50	40	10				22.0 ± 4.63	594 ± 238	8.0 ± 0.50	6.6–7.1
11	150–200	80–100	1.5–3.0	6	4	70	10				20	20.6 ± 6.97	225 ± 70	8.2 ± 0.51	6.5–7.1
12	30–40	3–10	0.3–1.0	10	3	60	40					20.7 ± 5.03	342 ± 79	10.5 ± 0.84	6.5–7.0
13	150–200	130–150	1.5–3.0	6	4	90					10	21.8 ± 5.79	320 ± 35	8.6 ± 0.80	6.7–7.1
14	250–300	200–250	0.5–2.0	1	5	80	10				10	21.0 ± 0.14	360 ± 99	7.5 ± 0.68	6.7–7.1
15	40–50	20–30	1.0–1.2	8	3	90	10					20.7 ± 4.74	319 ± 51	8.4 ± 0.90	6.7–7.1
16	90–120	5–30	0.3–1.2	9	3	60	20			10	10	20.4 ± 4.75	335 ± 51	7.5 ± 0.65	6.5–7.0
17	150–200	80–100	1.5–3.0	5	4	70	20				10	21.8 ± 4.94	277 ± 65	9.1 ± 0.46	6.8–7.3

Note: M: Mud (<0.1 mm); S: Sand (0.1–2 mm); G: Gravel (2–16 mm); P: Pebble (16–64 mm); C: Cobble (64–256 mm); B: Boulder (256 < mm) – modified Cummins (1962).

### Ichthyofauna

From the Dongjin River drainage system, a total of 4903 individuals of 53 species belonging to 14 families were found (Table 2). From the uppermost-stream station (St. 1), only seven species were sampled. From the upper mid-stream stations (St. 2–5), 16–21 species were found. Moving toward the lower-stream stations, from St. 6 to St. 7, and 11, the number of sampled species was decreased to 13, 11, and 10 species, respectively. A total of 18 species were collected at the lowest station (St. 14). In the tributaries, 10–21 species were found at the Jeongeup Stream (St. 8–10), 8–21 species were found at the Gobu Stream (St. 12–13), and 10–14 species were found at the Wonpyeong Stream (St. 15–17).

**Table 2.** List of fish species and the number of fish collected in the Dongjin River drainage system, Jeollabuk-do, Korea, from spring to fall, 2014.

Scientific name	Stations																	Total	RA^a^ (%)	Remarks
	1	2	3	4	5	6	7	8	9	10	11	12	13	14	15	16	17			
Order Petromyzontiformes																				
Family Petromyzonidae																				
* Lethenteron reissneri*				2	8													10	0.20	En-II, L
Order Anguilliformes																				
* *Family Anguillidae																				
* Anguilla japonica*														1				1	0.02	C
Order Cypriniformes																				
* *Family Cyprinidae																				
* Cyprinus carpio*								2	1		1	1		1				6	0.12	
*Carassius auratus*		1	3		6	8	12	22	8	11	4	14	2	6	1	15	4	117	2.39	
*Carassius cuvieri*									11		2	2	5	25	53	16	13	127	2.59	Ex
*Rhodeus ocellatus*									2									2	0.04	
*Rhodeus uyekii*		5							4			10						19	0.39	E
*Rhodeus notatus*												9						9	0.18	
*Acheilognathus lanceolatus*						6	1			3		7				7		24	0.49	E
*Acheilognathus koreensis*				9														9	0.18	
*Acheilognathus rhombeus*															2		8	10	0.20	
*Acheilognathus chankaensis*												12	3	8		4	2	29	0.59	
*Pseudorasbora parva*					4				11		19	35	30	17	13	21		150	3.06	
*Pungtungia herzi*					3													3	0.06	
*Sarcocheilichthys nigripinnis morii*														2				2	0.04	E
*Gnathopogon strigatus*																4		4	0.08	
*Squalidus gracilis majimae*		35		12					40						7	3		97	1.98	E
*Squalidus chankaensis tsuchigae*							7			17		2		75			2	103	2.10	E
*Hemibarbus labeo*										2	7			11				20	0.41	
*Hemibarbus longirostris*		13	4	4		12	15		11									59	1.20	
*Abbottina springeri*		22	24	35			52	2	255	1				1			1	393	8.02	E
*Abbottina rivularis*																	2	2	0.04	
*Pseudogobio esocinus*		12	32	15	25	85	58		37	2	22	26		4		39	6	363	7.40	
*Microphysogobio yaluensis*		25			4	12	140		58	1	25	4		1				270	5.51	E
*Microphysogobio jeoni*														2				2	0.04	E
*Aphyocypris chinensis*																2		2	0.04	
*Rhynchocypris oxycephalus*	18	140	56		5	2		6										227	4.63	
*Zacco koreanus*	85	105	100	130	68	10		21										519	10.59	E
*Zacco platypus*		131	66	60	64	43	27	20	265	140	45	16			17	19		913	18.62	
*Opsariichthys uncirostris amurensis*										10				5				15	0.31	
*Hemiculter eigenmanni*	2										2	80	17	45	13		16	175	3.57	E
*Erythroculter erythropterus*														50				50	1.02	
* *Family Balitoridae																				
* Lefua costata*								3										3	0.06	
* *Family Cobitidae																				
* Misgurnus anguillicaudatus*	4	13	5	5	2	1			1			6			3	3		43	0.88	
* Misgurnus mizolepis*		7	1															8	0.16	
* Iksookimia koreensis*	1	45	4	7			1	5	6									69	1.41	E
* Iksookimia longicorpa*			5	7	4													16	0.33	E
* Cobitis lutheri*					1	3			60			26			52	37		179	3.65	
* Cobitis tetralineata*		8		9	2													19	0.39	E
* Cobitis lutheri-tetralineata* hybrid		130	53	80	47	18	5											333	6.79	
Order Siluriformes																				
* *Family Siluridae																				
* Silurus microdorsalis*		1																1	0.02	E
* Silurus asotus*												4						4	0.08	
* *Family Bagridae																				
* Pseudobagrus fulvidraco*		1							5									6	0.12	
* *Family Amblycipitidae																				
* Liobagrus somjinensis*		6	2	5	2													15	0.31	E
Order Mugiliformes																				
* *Family Mugilidae																				
* Chelon haematocheilus*														13				13	0.27	
Order Beloniformes																				
* *Family Adrianichthyidae																				
* Oryzias sinensis*									5			11						16	0.33	
Order Perciformes																				
* *Family Centropomidae																				
* Coreoperca herzi*									3									3	0.06	E
* *Family Centrachidae																				
* Lepomis macrochirus*								8				18	1					27	0.55	Ex
* Micropterus salmoides*								3	2	7	5	1	2	5			56	81	1.65	Ex
* *Family Odontobutidae																				
* Odontobutis platycephala*		4	15	3	3	2		2	4									33	0.67	E
* Odontobutis interrupta*												5						5	0.10	E
* *Family Gobiidae																				
* Rhinogobius giurinus*												5				8		13	0.27	
* Rhinogobius brunneus*	30	89	13	30		9	5	4	17				5		3	6	3	214	4.36	L
* Tridentiger brevispinis*	11	30	6	20	3													70	1.43	
* *Number of species	7	21	16	17	17	13	11	12	21	10	10	21	8	18	10	14	11	53		
* *Number of individuals	151	823	389	433	251	211	323	98	806	194	132	294	65	272	164	184	113	4903		

Note: En-II: endangered species rank II; E: endemic species; A: amphidromous species; C: catadromous species; L: land-locked species; Ex: exotic species.

^a^RA: relative abundance (%).

The most dominant species in the Dongjin River drainage system was *Zacco platypus* (18.6%), followed by *Zacco koreanus* (10.6%), *Abbottina springeri* (8.0%), *Pseudogobio esocinus* (7.4%), *Cobitis lutheri*-*tetralineata* hybrid (6.8%), *Microphysogobio yaluensis* (5.5%), *Rhynchocypris oxycephalus* (4.6%), *Rhinogobius brunneus* (4.4%), *Cobitis lutheri* (3.7%), *Hemiculter eigenmanni* (3.6%), *Pseudorasbora parva* (3.1%), and so on.

Among the species found in the Dongjin River drainage system, *Lethenteron reissneri* that was designated as level II endangered species by the Ministry of Environment of Korea was found. Moreover, 18 species (34.0%) endemic to Korea were found along with three exotic species (5.7%) *Carassius cuvieri*, *M. salmoides*, and *L. macrochirus*, one (1.9%) catadromous species *Anguilla japonica*, and two (3.8%) land-locked species *L. reissneri* and *R. brunneus*.

### Similarity analysis

**Figure 2.**
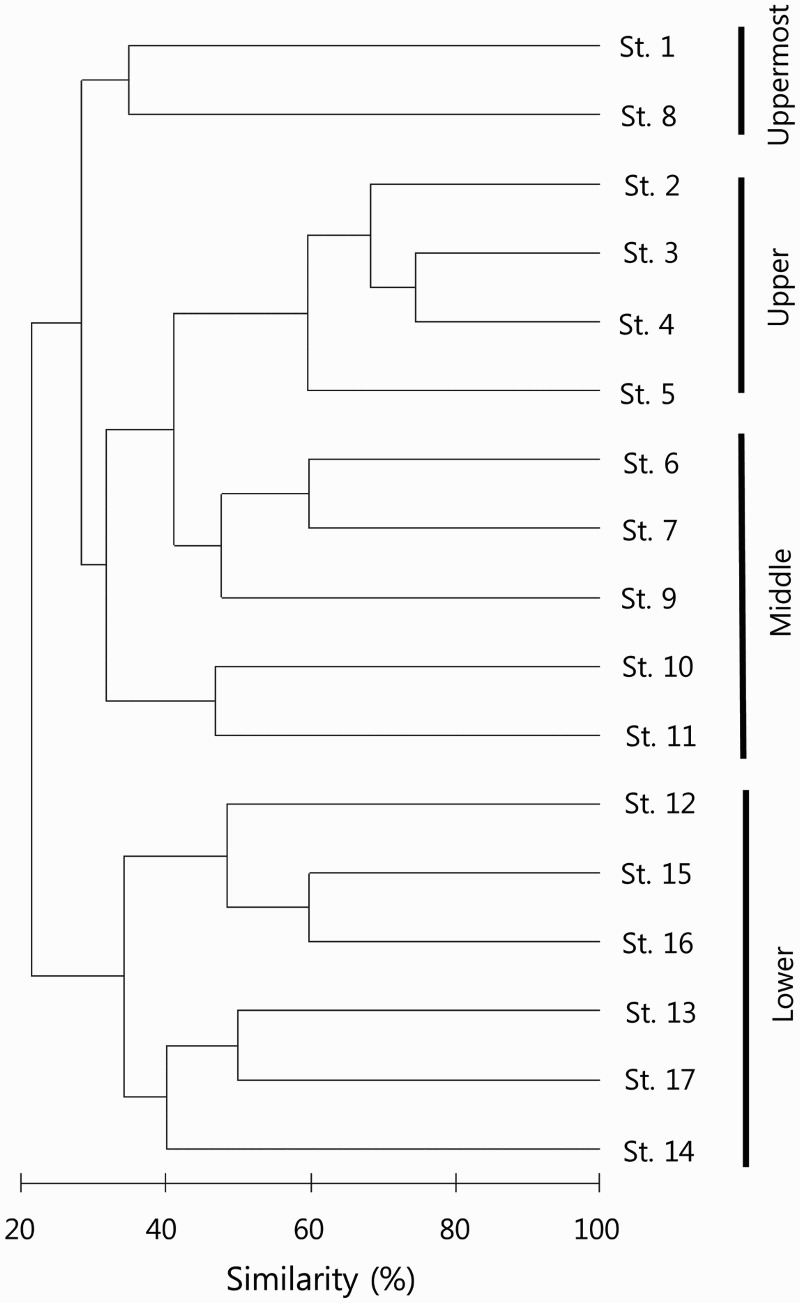
Dendrogram based on cluster analysis of similarity index of fish community in the sampling stations of the Dongjin River drainage system, Jeollabuk-do, Korea, 2014.

Similarity indices of species composition among different sites were calculated with the list of species and the abundance of each species at each site. Generally, uppermost (St. 1, 8), upper (St. 2–5), middle (St. 6–7, 9–11), and lower (St. 12–14) rive showed distinct separations except the upper and middle-streams (Figure 2).

### River health assessment

**Figure 3.**
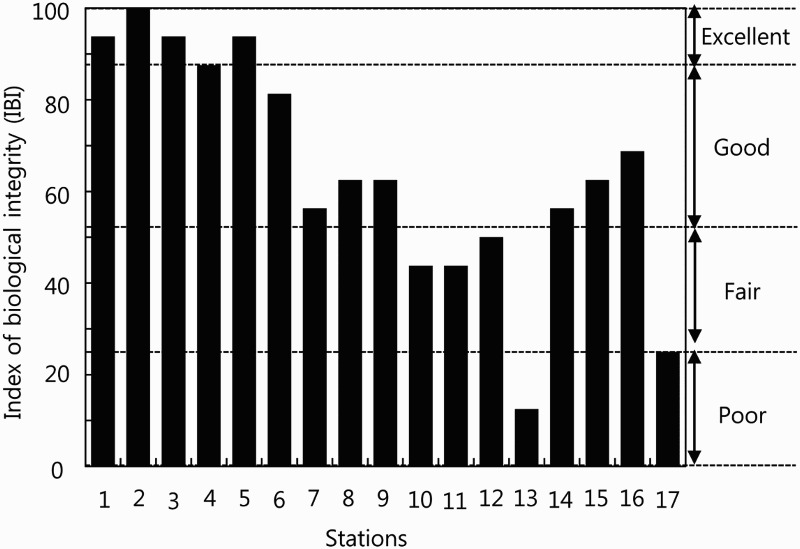
River health assessment for multivariate matrix index of biological integrity (IBI) in Dongjin River drainage system, Jeollabuk-do, Korea, 2014.

In the result of river health assessment, five stations were Excellent (87.5–100), seven stations Good (56.2–87.5), four stations Fair (25.0–56.2), and only one station Poor (0–0.25). The health of the upper-stream (St. 1–5) was very healthy with the score of Excellent. The health of the middle-stream (St. 6–11) was either in Good or Poor status. The health of the lower-stream was Good, Fair, or Poor. These results indicate that, as the water flows from the upper-stream to the lower-stream, the health of the river ecosystem deteriorates (Figure 3).

### Correlation

As a result of correlation analysis between physicochemical parameters and habitat characteristics of fishes, water temperature, conductivity, and salinity showed positive and significant (*p* < .01) correlation with each other. However, they showed negative correlation with the DO level (*p* < .05), and showed no correlation with pH (*p* > .05). The DO level showed positive correlation with SS (correlation coefficient *r* = 0.643, *p* < .01) and river health assessment (*r* = 0.508, *p* < .05) of habitat characteristics. On the other hand, conductivity showed negative correlation with SS (*r* = −0.628, *p* < .01) and river health assessment (*r* = −0.583, *p* < .05). Salinity also showed slight and negative correlation with SS (*r* = −0.488, *p* < .05).

## Discussion

**Figure 4.**
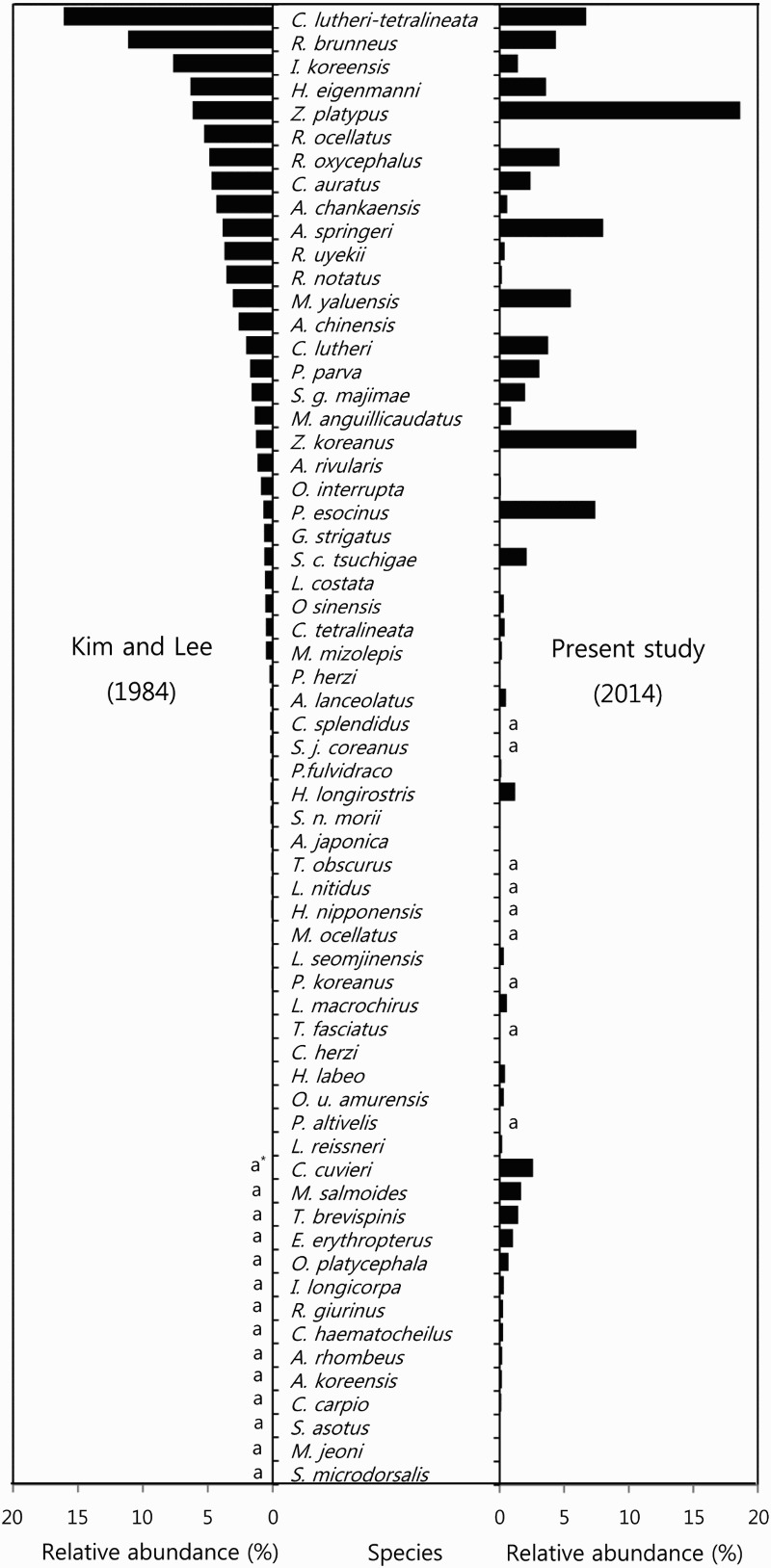
Relative abundance of fish species of the Dongjin River drainage system, Jeollabuk-do, Korea, from 1984 to 2014. *a: absence.

The present study investigated the changes of ichthyofauna in the Dongjin River drainage system in the past 30 years and explained faunal changes in the light of major influential environmental factors of that river. The Dongjin River has mixed impacts from diverse human activities unique to this river as well as general ones occurred in Korea in the past several decades. It is evident that the river is experiencing gradual river health deterioration from the upper-stream to the lower-stream. This tendency is consistent with the direction of increased human influence as discussed below: (i) construction of the Saemangeum dikes, (ii) construction of floodgates and weirs, (iii) dispersal of exotic species, (iv) water pollution, and (v) artificial water diversion tunnels (Figure 4).

### Ichthyofaunal changes in the past 30 years (1984–2014)

In the perspective of the number of species, Kim and Lee (1984) first reported 49 species belonging to 16 families from 12 sites using casting net and skimming net of the Dongjin River drainage system. Then Kim (2000) reported 50 species belonging to 14 families from 21 sites using casting net, skimming net, and fixed shore net. Later Kim et al. (2009) reported 30 species belonging to 6 families from 3 sites using casting net and skimming net of the Dongjin River. These previous results are generally similar to the 53 species belonging to 14 families from 17 sites found in this study. However, five species (*Microphysogobio jeoni*, *Erythroculter erythropterus*, *Iksookimia longicorpa*, *Silurus microdorsalis*, and *Chelon haematocheilus*) were observed in the present study for the first time. Moreover, 18 species composed of peripheral freshwater fish (6 species), Acheilognathinae (2 species), and others found previously were not found in this study. Additionally, a comparison of relative species abundance with that of Kim and Lee (1984) in which similar methods skimming and casting nets were used revealed sharp increases of some epipelagic, benthic, and exotic fish species. However, species that showed a rapid decrease in their relative abundance were some benthic and Acheilognathinae fishes. Therefore, in the last 30 years, fishes of the Dongjin River changed notably in the list of species and their relative abundance.

### The saemangeum sea-wall project

The Dongjin River estuarine environment has immensely changed due to the construction of Saemangeum sea wall (construction 1991–2006), the largest sea wall (33 km in total length) constructed in the world for the purpose of land reclamation. Since the progress of the project, a series of studies have reported the rapid changes of ichthyofauna around the sea wall (Lee et al. 2007; Park et al. 2013). Before the construction of dikes, estuary of the Dongjin River water system at the inner side of the Saemangeum sea wall was mudflat with brackish water. However, after the construction of dikes, the mudflat was transformed to an area covered by freshwater (Park et al. 2013). The estuary of Dongjin River was a known habitat for many peripheral freshwater fishes such as *Leiocassis nitidus*, *Trachidermus fasciatus*, *Synechogobius hasta*, *Periophthalmus modestus*, and *Mugil cephalus* in the past (Kim & Lee 1984; Kim 2000; Kim et al. 2009). In our study, however, the estuarine site (St. 14) turned out to be a pure freshwater zone with a salinity of 0.13–0.23‰ with primary freshwater fishes.

### Construction of floodgates and weirs

Large floodgates and numerous small weirs can influence the faunal composition. Several floodgates were constructed at the lower end of Dongjin River drainage to block the inflow of seawater and secure agricultural water (Figure 1), including Baegsan floodgate (built in 1978) in the main river, Hajang floodgate (1979) in the Gobu Stream, and Haechang floodgate (1978) in the Wonpyeong Stream. These constructions transformed the lower and middle regions into lentic water. For instance, 30 years ago, St. 11 was inhabited by 19 species of riffle, lentic, and benthic fishes including *M. yaluensis*, *R. uyekii*, *C. lutheri-tetralineata* hybrid, and so on (Kim & Lee 1984). However, it is now inhabited by only 10 species mostly found in lentic water such as lakes. In addition, large and small weirs (531) are still being built continuously along the mainstream and its tributaries since 1945 to supply agricultural water (Jang et al. 2010). The construction of weirs was followed by decline of riffles but increase of lentic waters that can also affect fish habitation.

### Introduced fishes from the Seomjin River through artificial water diversion tunnels

Along with water influx from the Seomjin River to Dongjing River via water diversion tunnels built about 80 years ago, unidirectional inflow of fishes toward the Dongjin from the Seomjin has been reported. For instance, Kim and Lee (1984) have reported the introduction of six species (*Hypomesus nipponensis*, *H. eigenmanni*, *Pseudobargrus koreanus*, *Liobagrus somjinensis*, *Cobitis tetralineata*, and *L. macrochirus*). More recently, four species (*Acheilognathus koreensis*, *Iksookimia longicorpa*, *Tridentiger brevispini*, and *M. salmoides*) were introduced from the Seomjin to Dongjin River. A notable introduction is the spined loach *C. tetralineata* that is endemic to the Seomjin River because of its widespread hybridization with native congeneric species (Kim & Lee 1984, Kim & Yang 1993, Kwan et al. 2014). In 2014, mating experiments of *C. tetralineata* and *C. lutheri* by Kwan et al. (2014) revealed that they are not reproductively isolated and their hybrid ratio in the wild was as high as 93%. In conclusion, dilution of the gene pool of native *C. lutheri* by introduced non-native *C. tetralineata* was suggested through a series of population genetic analyses based on multi-locus microsatellites makers and nuclear gene sequences (Kwan et al. 2014).

### Exotic species

Exotic species have been consistently introduced to Korea for food and recreational purposes (Kim et al. 1996). By 2011, there were 146 exotic species in Korea (Bang et al. 2011). Nowadays, exotic species are still being introduced and dispersed to the Dongjin River drainage system. Bluegill *L. macrochirus* was the first exotic species reported from the Dongjin River by Kim and Lee (1984). Later, Kim (2000) confirmed four exotic species (*M. salmoides*, *L. macrochirus*, *C. cuvieri*, and *Cyprinus carpio* (Israeli type)) as inhabitants of the Dongjin River drainage system. In this study, three exotic species (*M. salmoides*, *L. macrochirus*, and *C. cuvieri*) were found, while *C. carpio* (Israeli type) was not detected. The existence of these three species in the past and present reveals that there is a tendency of dispersal and gradual expansion of population size from mid-lower region to other regions (Table 2). This tendency seems to be triggered by the formation of lentic water by floodgates and weirs that *M. salmoides*, *L. macrochirus*, and *C. cuvieri* favor (Kawanabe & Mizuno 1989; Kim & Park 2007). Particularly, *M. salmoides* has been reported to cause rapid decreases in population size of endemic and indigenous fish species (Maezono et al. 2005; Jang et al. 2006). The dispersal and high predation pressure of *M. salmoides* on native fishes might have caused the apparent decrease in population size of small fish species in the mid-lower region of Dongjin River.

### Water pollution and river health assessment

DO level, pH, conductivity, and other chemical factors can directly affect fish habitat and distribution (Moyle & Cech 2000). Deterioration of water quality is known to decrease the number of SS preferentially. Furthermore, severely polluted water has been often reported to trigger mass mortality (Haslouer 1979). In this study, the water quality of upper mid-streams (St. 1–8) was good. However, as mid-lower streams joined the Jeongeup Stream, which is heavily polluted by the influx of domestic and industrial wastes from Jeongeup city (St. 10), the water quality becomes worse. Past water quality data of Jeongeup city is not available and therefore not comparable directly with the present data. However, the industrial complex, which affects the water quality the most, was constructed serially, one in 1981 and four from 1986 to 1995. From this, water quality of Jeongeup Stream is assumed to be good until the mid-1980s, but deteriorated greatly as the industrial complex size increased rapidly in the late 1980s. In this study, the DO level and conductivity turned out to be correlated with sensitive and tolerant species. SS are easily affected by environmental changes. They were found to inhabit the upper mid-streams (St. 1–8) where the DO level was high but the conductivity was low. However, in the mid-lower section where water quality deteriorated, the number of SS was decreased, resulting in the changes of ichthyofaunal composition in the last 30 years.

Fish takes an important position in aquafauna, and is used as one of criteria along with attached algae, benthic intertebrate, and waterside vegetation for assessing the health of Aquafauna and rivers in Korea (MEK 2007). In this study, we applied a derivative of this approach developed by the Ministry of Environment of Korea and appropriately incorporated realistic features of Korean rivers and fishes for the assessment of river health at 17 stations in the river. Five stations were classified as ‘Excellent’ and seven stations were classified as ‘Good’. Other stations were in vulnerable state.

### Conservation and management of the Dongjin River

For the systematic conservation and management of the Dongjin River, the strategies should be set up separately for each region. For the upstream of Dongjin River, consistent monitoring of the fish from the Seomjin River is necessary. For Jeongeup Stream, influx of domestic and industrial waste from the city should be controlled and the plans to improve the water quality are needed. Moreover, a plan is needed to control alien species which are rapidly increasing in mid and downstream of the Dongjin River. In particular, largemouth bass is an aggressive predator and its further dispersal needs to be prevented. Estuary environment of the Dongjin River is continuously changing caused by Saemangeum sea-wall construction, so consistent observation leading to its management is required.
